# MICA and NKG2D: Is There an Impact on Kidney Transplant Outcome?

**DOI:** 10.3389/fimmu.2017.00179

**Published:** 2017-02-27

**Authors:** Matilde Risti, Maria da Graça Bicalho

**Affiliations:** ^1^LIGH – Immunogenetics and Histocompatibility Laboratory, Federal University of Paraná, Curitiba, Brazil

**Keywords:** transplantation, kidney, allograft, MICA, MICA-129, NKG2D, LNK1, HNK1

## Abstract

This paper aims to present an overview of MICA and natural killer group 2 member D (NKG2D) genetic and functional interactions and their impact on kidney transplant outcome. Organ transplantation has gone from what can accurately be called a “clinical experiment” to a routine and reliable practice, which has proven to be clinically relevant, life-saving and cost-effective when compared with non-transplantation management strategies of both chronic and acute end-stage organ failures. The kidney is the most frequently transplanted organ in the world (transplant-observatory^1^). The two treatment options for end-stage renal disease (ESRD) are dialysis and/or transplantation. Compared with dialysis, transplantation is associated with significant improvements in quality of life and overall longevity. A strong relationship exists between allograft loss and human leukocyte antigens (HLA) antibodies (Abs). HLA Abs are not the only factor involved in graft loss, as multiple studies have shown that non-HLA antigens are also involved, even when a patient has a good HLA matche and receives standard immunosuppressive therapy. A deeper understanding of other biomarkers is therefore important, as it is likely to lead to better monitoring (and consequent success) of organ transplants. The objective is to fill the void left by extensive reviews that do not often dive this deep into the importance of MICA and NKG2D in allograft acceptance and their partnership in the immune response. There are few papers that explore the relationship between these two protagonists when it comes to kidney transplantation. This is especially true for the role of NKG2D in kidney transplantation. These reasons give a special importance to this review, which aims to be a helpful tool in the hands of researchers in this field.

## Introduction

Genetic diversity is the hallmark of MHC genes (1). The main antigenic barrier to transplantation is molecules, which are polypeptide products of a cluster of genes known, in humans, as human leukocyte antigens (HLA). In addition, a family of highly glycosylated MHC-encoded molecules, the *MHC class I chain-related (MIC)* genes, has been identified (2) as a second lineage of mammalian MHC I genes, which could constitute an antigenic barrier to transplantation as well (3). The MIC molecules possess a low degree of homology to other *MHC class I* encoded genes and interact with both T-cell and natural killer (NK)-cell receptors (2). MIC proteins act as ligands for NK cells, γδ T cells, and αβ CD8^+^ T cells, which express natural killer group 2 member D (NKG2D) ligand (4). The importance of the MICA protein in kidney transplantation has been acknowledged in recent years, and the role they play in graft rejection has been intensely pursued.

## *MICA* Gene: Structure, Polymorphisms, and Function

**Figure 1:**
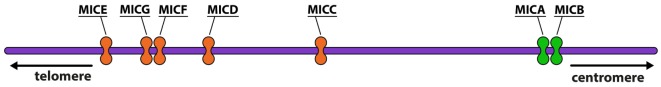
**Representation of *MHC class I chain-related (MIC)* genes**. The functional genes are represented in green and the pseudogenes are in orange (image by Matilde Risti).

The *MIC* gene family consists of seven members (*MICA–MICG*) (Figure 1), five of which are pseudogenes, and two, *MICA* and *MICB*, of which are functional (5, 6). *MICA* and *MICB* are the most divergent members of the human MHC-encoded class I genes identified to date, having an average of 19, 25, and 35% similarity in the extracellular α1, α2, and α3 domains, respectively, to those of other MHC α-polypeptides (7).

The *MICA* gene is located 46.4 kb centromeric to *HLA–B* on the short (p) arm of chromosome 6 at position 21.33 (3).

*MICA* and *MICB* have been shown to differ in the transcriptional control regions from common HLA class I genes. *MICA/B* genes lack the prototypic MHC class I gene promoter regulatory elements, the SXY module [heterotrimeric X-box-binding factor—regulatory factor X; X2-box-binding factor—cyclic-AMP-responsive-element-binding protein; Y-box-binding factor—nuclear transcription factor Y (NF-Y); and an as-yet-unidentified S-box-binding factor]. In contrast, the regulatory promoter module of *MICA/B* contains heat shock elements resembling those of HSP70 genes, a CCAAT box that binds to nuclear transcription factor Y (NF-Y), and a GC box that binds to Sp1, Sp3, and Sp4 transcription factors (8).

There are 12 known possible haplotypes of *MICA* 5′ promoter regions, including a null haplotype due to a deletion of the entire *MICA* gene (*MICA*-P12), which are more densely distributed in both ends compared to the central portion of 5′ promoter (8, 9).

**Figure 2:**
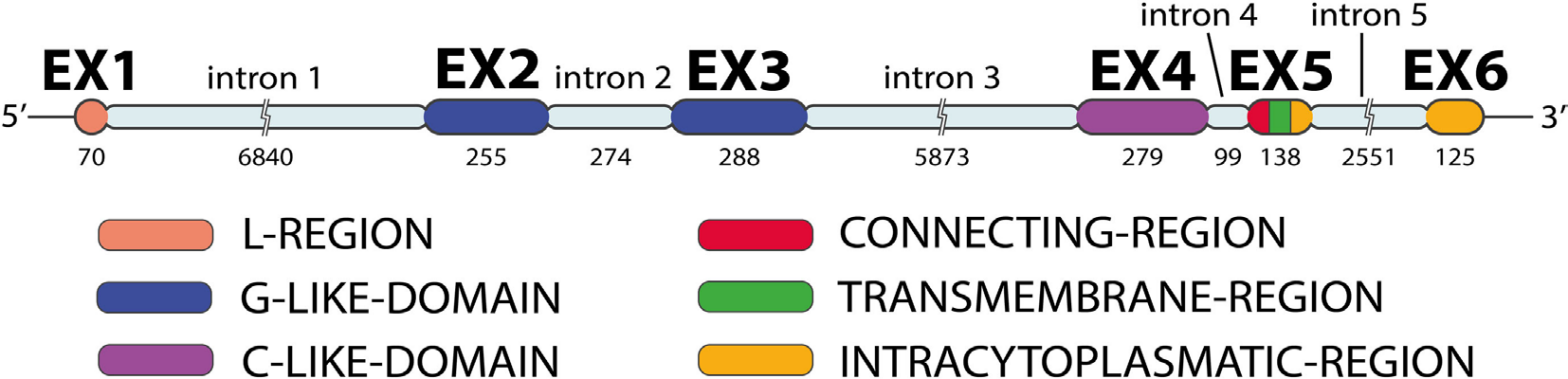
***MICA* gene exon–intron organization**. The *MICA* gene has five introns and six exons (image by Matilde Risti).

*MICA* has six exons separated by five introns (Figure 2): exon 1 encodes the leader peptide, exons 2–4 encode three extracellular globular domains, exon 5 encodes the transmembrane domain, and exon 6 encodes the cytoplasmatic tail (6, 10). An intron of 6,840 bp follows exon 1 and is unusually large for a class I gene. The remainder of the *MICA* gene has a quite similar organization to classical class I genes, except for the presence of a relatively long intron 5 and the fusion of the cytoplasmic tail and 3′ UTR sequence in a single last exon (11).

It is considered that *MICA* gene has a codominant expression, and the presence of heat shock elements within the promoter suggests that MICA transcription is indued under stress conditions, and that therefore the MICA protein functions as an indicator of cell stress (11–13). The first intron of the *MICA* gene contains an NFkB-binding site that binds p65 (RelA)/p50 heterodimers and p50/p50 homodimers of the NFkB transcription factor family. The role of the proximal −130 bp NFkB site was reported as necessary and sufficient for transcriptional transactivation of *MICA* in response to TNFα in primary endothelial cells (ECs) (14).

Gene transcription isoforms are mRNAs transcribed from the same locus that differs in their transcription start sites and/or untranslated regions or protein coding DNA sequences (CDSs) also producing different protein isoforms. The alternative splicing of *MICA* leads to the formation of four isoforms. Two of them were described by Zou and Stastny (15) (MICA isoforms 1 and 2), and they did not appear to be tissue specific.

*MICA* isoform 1 (1*001) is the longest isoform, derived from the MICA*001 allele. *MICA* isoform 2 (1*008:01) is a variant isoform derived from the MICA*008:01 allele that contains a four-nucleotide insertion (rs9279200), which causes a frameshift mutation and subsequent truncation of the CDS, compared to isoform 1 (allele MICA*001) (15). The other two isoforms of *MICA*, isoforms 3 and 4, are described only in the *ncbi.nih.gov/gene*^2^ website. *MICA* isoform 3 is, like isoform 2, encoded by the MICA*008:01 allele; however, it is shorter than isoform 2 at the N-terminus, containing an alternate 5′ exon, differences in the 5′ UTR, and lacking a portion of the 5′ coding region, with translation being initiated from a downstream in frame start codon. *MICA* isoform 4 contains an alternate 5′ exon and uses an alternate splice site in an internal exon. It differs in the 5′ UTR, lacks a portion of the 5′ coding region, and initiates translation from an alternate start codon, compared to variant 1 (MICA*008:01 allele). Isoform 4 has a distinct and shorter N-terminus, compared to isoform 2.

The *MIC* genes are transcribed in keratinocytes, ECs, fibroblasts, monocytes, epithelial cell lines and epithelial tissues of cell lines, and freshly isolated cells (2, 16) and are not usually transcribed in CD4^+^ T cells, CD8^+^ T cells, and CD19^+^ cells (17). MIC protein is only expressed on the cell surface of freshly isolated ECs, fibroblasts (17), and gastric epithelium (12). MIC protein acts as a ligand for NK cells, γδ T cells, and αβ CD8^+^ T cells, which express NKG2D ligand (NKG2DL) (4).

### *MICA* Polymorphism and -129Met/Val Dimorphism

Bahram et al. (3) first described MICA*01 to MICA*05 alleles with a total of 18 nucleotide substitutions resulting in 14 amino acid changes in the final polypeptide. Fodil et al. (7) described the alleles, MICA*06 to MICA*16, with nine nucleotide substitutions and eight amino acids changes. One year later, Mizuki et al. (18) showed a variable number of trinucleotide GCT repeats that encode 4, 5, 6, 7, 9, or 10 alanine (A, Ala). The short tandem repeats or microsatellite alleles were labeled as *A4, A5, A6, A7, A8, A9*, and *A10*. There is also an *A5.1* allele that contains five triplet repeats of GCT plus an additional guanine nucleotide insertion (GGCT). This insertion causes a frameshift mutation leading to a premature intradomain stop codon within the transmembrane region, which deletes the MICA cytoplasmic tail. The A4, A5, A6, A7, A8, A9, A10, and A5.1 sizes are, respectively, 179, 182, 185, 194, and 183 bp (18–20). At the time of writing (October 2016) hla.alleles.org^3^ reports 105 *MICA* alleles, 2 of which considered null, result in 82 different MICA proteins. All *MICA* alleles from *001 to *087 producing different proteins and their nucleotides variations on exons 2–6 are shown in Table 1.

**Table 1:** Nucleotide variations on exons 2–6 for *MICA* alleles from *001 to *087.

EXON 2 α1		
CODON 6	CTG (LEU)	CGC (ARG) CTC (PRO)
CODON 14	TGG (TRP)	GGG (GLY)
CODON 23	CTC (LEU)	GTT (LEU)
CODON 24	ACT (THR)	GCT (VAL)
CODON 26	GTA (VAL)	GGA (GLY)
CODON 36	TGT (CYS)	TAT (TYR)
CODON 38	AGG (ARG)	AGC (SER)
CODON 39	CAG (GLN)	TAG (Stop)
CODON 55	GGA (GLY)	GGC (GLY)
CODON 56	AAT (ASN)	AAC (ASN)
CODON 64	AGA (ARG)	AAG (ARG)
CODON 69	AAC (ASN)	AAT (ASN)
**EXON 3 α2**		
CODON 90	CTC (LEU)	TTC (PHE)
CODON 91	CAG (GLN)	CGG (ARG)
CODON 93	ATT (ILE)	ATG (MET)
CODON 102	AAC (ASN)	AGC (SER)
CODON 105	AAG (ARG)	AAG (LYS)
CODON 112	TAC (TYR)	TAT (TYR)
CODON 114	GGG (GLY)	AGG (ARG)
CODON 122	CTG (LEU)	GTG (VAL)
CODON 124	ACT (THR)	TCT (SER)
CODON 125	AAG (LYS)	GAG (GLU)
CODON 129	ATG (MET)	GTG (VAL)
CODON 130	CCC (PRO)	TCC (SER)
CODON 139	GCC (ALA)	GCA (ALA)
CODON 142	GTC (VAL)	ATC (ILE)
CODON 151	ATG (MET)	GTG (VAL)
CODON 156	CAC (HIS)	CTC (LEU) CGC (ARG)
CODON 169	CGG (ARG)	TGG (TRP)
CODON 173	AAA (LYS)	GAA (GLU)
CODON 174	TCC (SER)	TCT (SER)
CODON 175	GGC (GLY)	AGC (SER) GGT (GLY)
CODON 176	GTA (VAL)	ATA (ILE)
CODON 181	ACA (THR)	AGA (ARG)
**EXON 4 α3**		
CODON 190	CGC (ARG)	TGC (CYS)
CODON 191	AGC (SER)	AGT (SER)
CODON 193	GCC (ALA)	GCA (ALA)
CODON 198	ATT (ILE)	ATC (ILE)
CODON 205	TCT (SER)	TCC (SER)
CODON 206	GGC (GLY)	AGC (SER)
CODON 208	TAT (TYR)	TGT (CYS)
CODON 210	TGG (TRP)	CGG (ARG)
CODON 213	ACA (THR)	ATA (ILE)
CODON 215	AGC (SER)	ACC (THR)
CODON 221	GTA (VAL)	CTA (LEU)
CODON 230	TGG (TRP)	TCG (SER)
CODON 244	TGG (TRP)	TGA (Stop)
CODON 247	AAC (THR)	ACT (THR)
CODON 250	TGC (CYS)	CGC (ARG)
CODON 251	CAA (GLN)	CGA (ARG)
CODON 253	GAG (GLU)	AAG (LYS)
CODON 254	GAG (GLU)	AAG (LYS)
CODON 256	AAG (ARG)	AGT (SER) AAG (LYS)
CODON 265	GGG (GLY)	AGG (ARG)
CODON 268	AGC (SER)	GGC (GLY)
CODON 269	ACT (THR)	ATT (ILE)
CODON 271	CCT (PRO)	GCT (ALA)
**EXON 5 TM**		
CODON 295	CGT (ALA)	GCGT
CODON 304	TAT (TYR)	TAC (TYR)
CODON 306	CGT (ARG)	TGT (CYS)
**EXON 6**		
CODON 350	GAT (ASP)	GCT (ALA)
CODON 354	ACT (THR)	GCT (ALA)
CODON 359	GGC (GLY)	GGT (GLY)
CODON 360	GCC (ALA)	ACC(THR)

Codons are shown in the first column. The second column shows the triplets and their corresponding amino acids in the consensus sequence (MICA*001). The third column lists that triplet’s possible variations in other alleles compared with the consensus sequence. Amino acid substitutions in MICA on the three external protein domains (exons 2–4), on the transmembrane domain TM (exon 5) and carboxy-terminal cytoplasmic tail (exon 6). The G nucleotide insertion is represented in red in the exon 5 TM.

**Figure 3:**
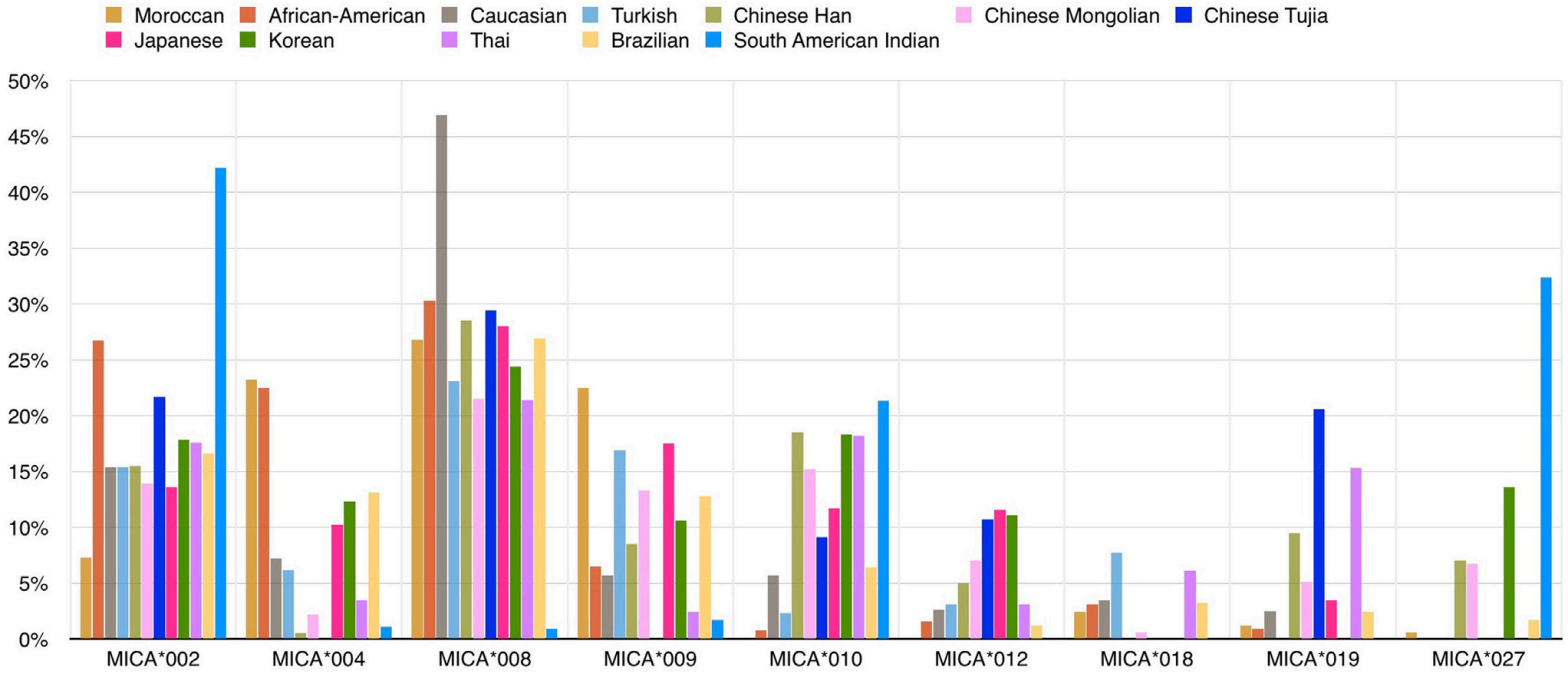
**Frequencies (%) of common *MICA* alleles within 12 populations**. The allele frequencies of nine *MICA* alleles are shown for 12 populations: Caucasoid (21–23), Korean (19), North-Eastern Thai (27), Japanese (13, 28), African-American (21, 29), South American Indian (25), Moroccan (30), Turkish (31), Brazilian (24, 32), Chinese Mongolian (33), and Chinese Tujia (34, 35) (image by Matilde Risti).

Several studies have documented *MICA* allele frequencies within different populations (Figure 3), and the frequency distribution varies between them. For example, the same group of three alleles (MICA*008, MICA*002, and MICA*004) accounts for more than 50% of the allele frequencies commonly found in several Caucasoid populations (21–24) but at the same time MICA*027’s frequency is extremely different in a comparison between the South American Indian and Caucasoid populations (25). Single high-frequency *MICA* alleles are each associated with more than one different *HLA-*B allele, but this pattern is not reciprocal. Most specific *HLA-B* alleles, including B*07:02 and B*08:01 variations, are usually linked to a single *MICA* allele. This pattern suggests that the *MICA* alleles had an earlier origin than major branches of *HLA-B* alleles (26).

The evolutionary history of *HLA-B* alleles is recognizable in the linkage relationship between *HLA-B* and *MICA* genes. The high degree of sequence similarity between three *HLA* alleles (B*35, B*53, and B*58) indicates that they were all generated from the same progenitor allele, and the observation that globally they are all linked to the MICA*002 allele further supports this conclusion. Specific *MICA* alleles also tend to associate with serological HLA-B groups. A rare exception can be found in B*44, whose two subgroups B*44:02 and B*44:03 have exclusive associations with MICA*008 and MICA*004 (26).

The *MICA-129Val/Met* dimorphism, caused by an SNP (rs1051792) at nucleotide position 454 (G>A) of the *MICA* gene is of particular interest. The substitution of valine (Val) for methionine (Met) at position 129 in the α2 domain of the MICA protein has been reported to affect NKG2D binding avidity (36–40). This dimorphism divides the *MICA* alleles into two groups (Table 2). In 2015, it has been observed that *MICA-129Met* alelles increased the risk of experiencing acute graft-versus-host disease. This effect could be the consequence on NKG2D signaling by MICA-129Met variant (40). In addition to this, it has been shown that the MICA-129 dimorphism may directly affect plasma membrane expression and shedding of MICA, and these functional effects might contribute to the numerous disease associations (41).

**Table 2:** **Dimorphism 129 Val/Met divides *MICA* alleles into two groups**.

Dimorphism 129 val/met divides *MICA* alleles in two groups

ATG (Met)	GTG (Val)
MICA*001, *002, *007, *011, *012, *014, *015, *017, *018, *020, *023, *025, *026, *029, *030, *031, *032, *034, *035, *036, *037, *038, *039, *040, *041, *042, *043, *045, *046, *047, *050, *051, *052, *055, *059, *060, *061, *068, *072, *075, *078, *079, *081, *083, *084, *086	MICA*004, *005, *006, *008, *009, *010, *013, *016, *019, *022, *024, *027, *028, *033, *044, *048, *049, *053, *054, *056, *057, *058, *062, *063, *064, *065, *066, *067, *069, *070, *073, *074, *076, *077, *080, *082, *085, *087

The most frequent alleles present in Figure 3 are shown in bold. The *MICA* alleles shown are from MICA*001 to MICA*087.^4^ “MICA*003:01” label has never been assigned to any sequence. MICA*021 sequence was renamed MICA*012:03 in August 2007. The sequence originally labeled MICA*071 was proven to contain errors and to be identical to MICA*017 (March 2013) (see text footnote 3).

### MICA Molecule

**Figure 4:**
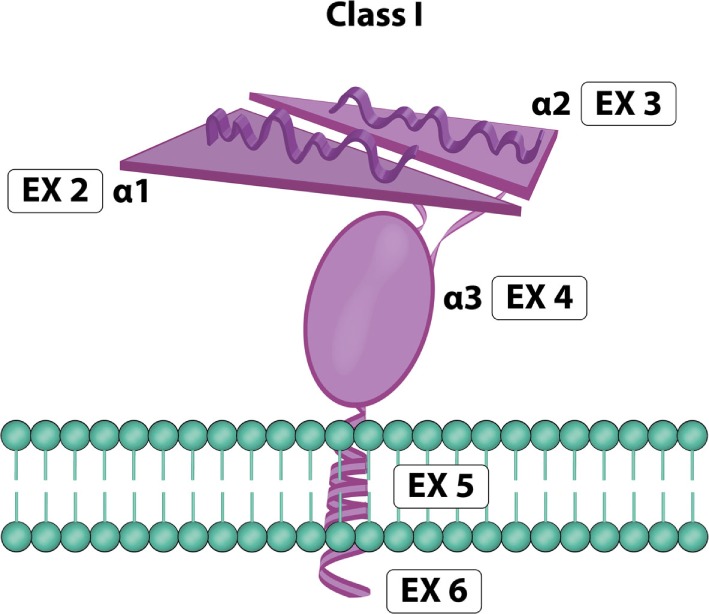
**MICA molecule**. Exon 1 encodes one leader peptide, exons 2–4 encode three extracellular globular domains, exon 5 encodes one transmembrane domain, and exon 6 encodes a cytoplasmic tail (image by Matilde Risti).

MICA is a highly glycosylated membrane-anchored cell surface protein composed of 383 amino acids (12). Unglycosylated MICA appeared less stable than those incorporating glycosylated MICA (36). Its expression has been reported on the surface of different cells and resembles the domain organization (Figure 4) of the α chain of MHC class I molecules (16, 42). MICA α chain does not bind β2-microglobulin and is independent of any transporter-associated protein. Attempts to identify peptides bound to MICA have been unsuccessful (10, 12). The crystal structure of MICA shows four distinct α helices arranged in an eight-stranded antiparallel β sheet. These helices in MICA roughly correspond to the two helices that define the peptide-binding groove in peptide-binding MHC class I proteins and homologs (42).

MICA is generally concentrated in lipid rafts and is *S*-acylated, similar to other lipid rafts-associated proteins. *In vitro* mutation of the S-acylation site, replacing a cysteine residue with a stop codon at aminoacid position 39, yields a truncated form of MICA, unable to activate NK cells (43).

The MICA molecule interacts with NK cells, γδ T cells, and αβ CD8^+^ T cells, which express NKG2D, a common activating NK cell receptor (4, 10, 44). NKG2D recognizes the human MICA protein in conjunction with a transmembrane signaling adaptor protein, DNAX-activation protein (DAP10) (4, 10).

It is noteworthy that the MICA molecule can also be recognized by γδ T cells with the TCR variable region V_δ_1 (4, 45–47).

Both types of receptors, V_δ_1TCR and NKG2D, can simultaneously recognize and bind to MICA on a V_δ_1 cell surface. There is close association between the tissue distribution of V_δ_1 cells and the physiological expression of MICA, as MICA affects V_δ_1 cell lineage development (46). In V_δ_1 _γδ_T cells, the strength of the binding between TCR and MICA is weaker than that between NKG2D and MICA. Although weak, TCR:MICA complexes show unusual stability after they are formed, with long half-lives. TCR and NKG2D receptors compete for binding to MIC ligands, and it has been suggested that initial interactions at the point of contact may be dominated by NKG2D:MIC binding events, which then give way to longer-lived γδ TCR:MIC complexes (47).

### Conclusions on MICA

The *MICA* gene is polymorphic, and it is in linkage disequilibrium with *HLA-B* genes. The MICA protein is expressed on the cell surface, and it is possibly the proteolytic cleavage of the α_3_ domain which in turn releases soluble MICA (sMICA). The MICA molecule does not present a peptide in its groove and can interact with the NKG2D receptor, which is the focus of the following paragraphs.

## NKG2D or *Killer Cell Lectin-Like Receptor K1 (KLRK1)* Gene: Structure, Polymorphisms, and Function

**Figure 5:**
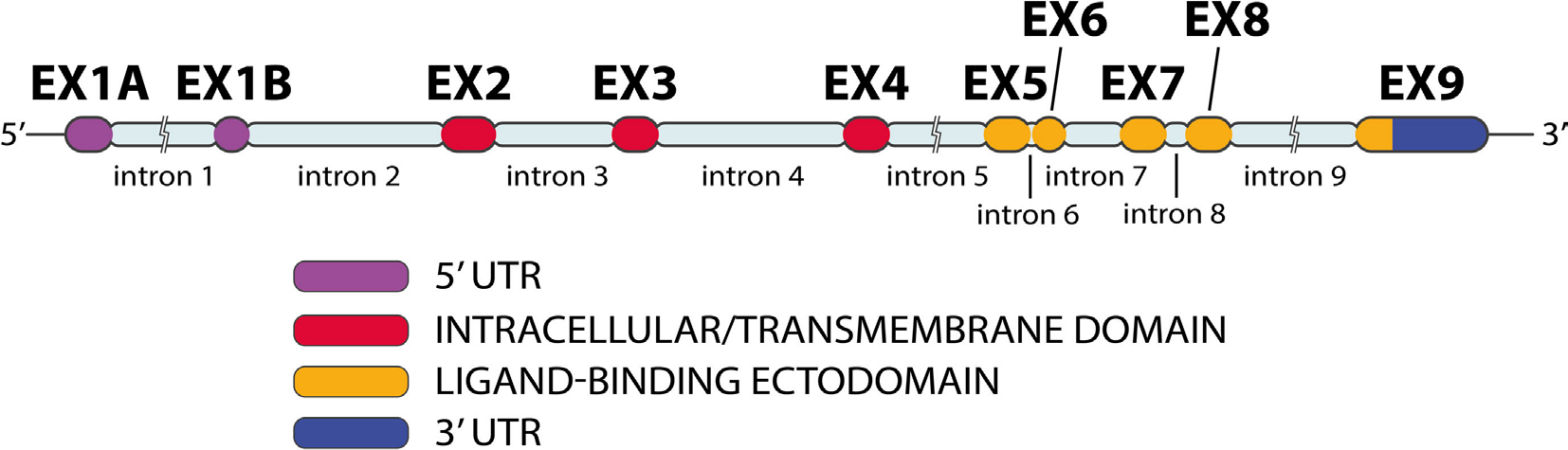
***NKG2D* gene exon–intron organization**. The *NKG2D* gene has 10 exons and 9 introns (image by Matilde Risti).

*NKG2D* gene, also known as KLRK1, is located in the natural killer complex (NKC) on chromosome 12 (42, 48, 49). Human *NKG2D* (Figure 5) has 10 exons (50). Exons 2–4 encode the intracellular/transmembrane domain; exons 5–8 encode the ligand-binding ectodomain, which is a membrane-bound domain protruding into extracellular space (50, 51). *NKG2D* has a low number of nucleotide variations (48). *NKG2D* appears to be conserved during evolution, with orthologs of *KLRK1* are present in the genome of all mammals, as well as in marsupials (4, 52).

Human *NKG2D* is expressed from at least three distinct alleles, and several gene transcription isoforms have been described, including an alternatively spliced variant that introduces a nonsense mutation resulting in a protein isoform that lacks the entire extracellular ligand-binding domain (53).

Hayashi et al. (54) evaluated the SNPs in the NKC gene region. They selected 20 SNPs with a >10% higher frequency in Caucasoid or Japanese populations (Table 3); these SNPs covered *CD94, NKG2D, NKG2F, NKG2E, NKG2A*, and *Ly49* genes. They selected 8 out of the 20 SNPs that were closely associated with natural cytotoxic activity, having *P* values <0.001. All these SNPs are located in the NKG2D gene region, except for rs1983526 that is located in the promoter region of the NKG2A gene. These eight SNPs were split into two groups: group 1 (rs1049174, rs2617160, rs2617170, rs2617171, and rs1983526) and group 2 (rs2255336, rs2246809, and rs2617169). All the SNP combinations of group 1/group 1 and group 2/group 2 revealed a strong linkage disequilibrium, with *r*^2^ values >0.9, whereas group 1/group 2 combinations showed much weaker linkage disequilibrium, with *r*^2^ values <0.5. This indicates that the five group 1 and three group 2 SNPs belong to two different haplotype blocks (NKG2D hb-1 and hb-2), each of which generates two major haplotypes associated with low (LNK) and high (HNK) natural cytotoxic activity phenotypes (Table 3) (54).

**Table 3:** **20 SNPs selected by Hayashi et al. in their study (54)**.

SNP ID	Variation	SNP ID	Variation	NKG2D hb-1	Low	High
rs3759272	G>T	rs2617170	T>C	**rs1049174**	C	G
rs2537752	T>A	rs2617171	C>G	rs2617170	C	T
**rs1049174**	G>C	rs1971939	C>G	rs2617171	C	G
rs2255336	A>G	rs1915319	A>G	rs1983526	C	G
rs2294148	G>A	rs4763525	G>A	rs2617160	T	A
				
rs2049796	A>C	rs3003	C>T	**NKG2D hb-2**	**Low**	**High**
				
rs2617160	A>T	rs1983526	C>G	rs2255336	G	A
rs7972757	A>G	rs10772285	G>C	rs2246809	G	A
rs2246809	A>G	rs1915325	G>A	rs2617169	T	A
rs2617169	T>A	rs2607893	C>T	

*Blue fields belong to group 1 and green ones represent group 2. Each of the different haplotype blocks (NKG2D hb-1 and hb-2) is split in low and high natural cytotoxic activity haplotypes. hb-1 and hb-2 may be successfully predicted knowing only rs1049174 (in bold)*.

A separate study on a European population confirmed that the NKG2D region haplotype associated with increased cancer susceptibility in the Japanese population also exists in Europeans at similar frequency. Therefore, the conclusions of the original study may also be applicable to this population (55).

### NKG2D: HNK1 and LNK1 Haploblocks

Several studies have demonstrated that high and low natural cytotoxic activity haplotype alleles (HNK1 or LNK1) belonging to NKG2D haplotype blocks 1 (hb-1) may be successfully predicted by only a single SNP (dbSNP: rs1049174) (54, 56, 57).

A study on Japanese individuals demonstrated that the *HNK1* haplotype is associated with a greater activity of NK cells in the peripheral blood and a lower prevalence of cancers originating from epithelial cells (58). Espinoza et al. showed an association between the *NKG2D-HNK1* haplotype (haplotype frequency, 61%) in bone marrow donors and a significantly reduced transplant-related mortality and better overall survival for unrelated donors of HLA-matched myeloablative bone marrow recipients with standard-risk disease (58).

**Figure 6:**
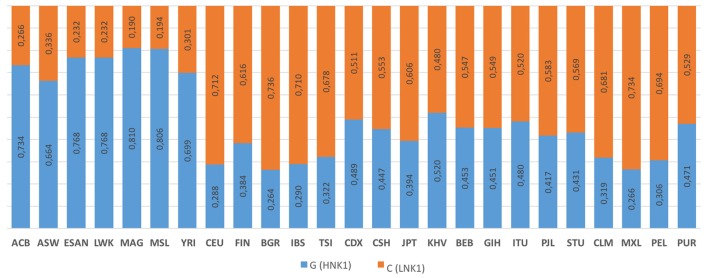
**1000 Genomes frequency for the G>C alleles (NKG2D hb-1) (see Footnote 5)**. The population represented are African Caribbean in Barbados (ACB), African Ancestry in Southwest US (ASW), Esan in Nigeria (ESAN), Luhya in Webuyu Kenya (LWK), Mandinka in Degambia (MAG), Mende in Sierra Leone (MSL), Yoruba in Ibadan Nigeria (YRI), Utah Residence with Northern and Western European Ancestry (CEU), Finnish in Finland (FIN), British in England and Scotland (BGR), Iberian populations in Spain (IBS), Toscani in Italy (TSI), Chinese Dai in Xishuangbanna, China (CDX), Han Chinese in Bejing, China (CSH), Japanese in Tokyo, Japan (JPT), Kinh in Ho Chi Minh City Vietnam (KHV), Bengali in Bangladesh (BEB), Gujarat Indian in Houston Texas (GIH), Indian Telegu in the UK (ITU), Punjabi in Lahore Pakistan (PJL), Srilankan Tamil in the UK (STU), Colombian in Medellin Colombia (CLM), Mexican Ancestry in Los Angeles, California, USA (MXL), Peruvian in Lima Peru (PEL), and Puerto Rican in Puerto Rico (PUR).

The rs1049174 distribution for 25 populations (Figure 6) is reported on the 1,000 genomes website.^5^ HNK is reported to be associated with the rs1049174 (G) allele, and LNK with rs1049174 (C) (54, 56).

### NKG2D Protein

The NKG2D is a member of a C-type lectin-like family receptor called CD94/NKG2 (42). Despite its inclusion in the NKG2 family, NKG2D displays only limited sequence similarity to other members of the NKG2 family of NK cell surface receptors (NCRs) and CD94 and forms homodimers, rather than heterodimers, with CD94, as do other NKG2 NCRs (42).

Natural killer group 2 member D is a transmembrane-anchored receptor expressed as a disulfide-linked homodimer on the cell surface, with a molecular weight of ~42 kDa (42).

**Figure 7:**
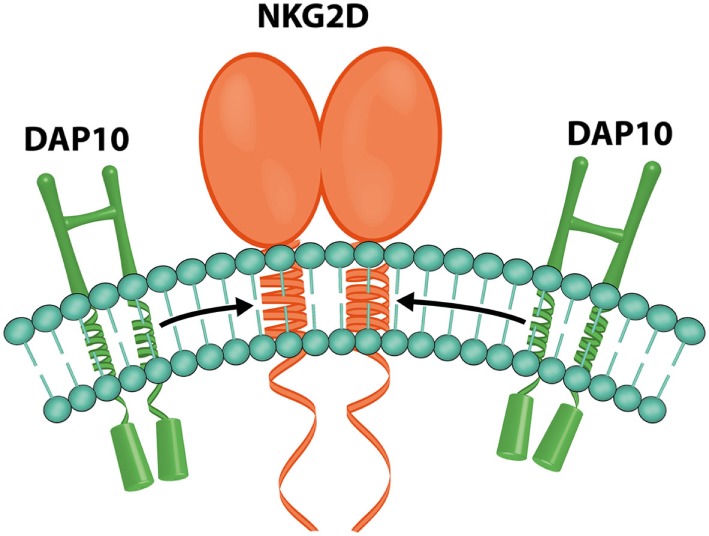
**NKG2D and DAP10**. Representation of the hexametric structure formed by one NKG2D and two DAP10 homodimers (image by Matilde Risti).

In humans, each NKG2D homodimer (Figure 7) associates with two DAP10 homodimers to form a hexameric structure (59), which can signal by recruitment of phosphatidylinositol 3-kinase (36).

Human NKG2DLs are MICA and MICB, and a group of glycosylphosphatidylinositol-bound surface molecules including UL16 binding protein(ULBP)-1, -2, -3, and -4 (6), RAET1G (or ULBP5), and RAET1L (or ULBP6) (60), which share about 25% identical amino acids in their α_1_α_2_ domains that are variably scattered throughout the aligned sequences without discernible patterns of sequence conservation (36).

Signals triggered by the NKG2D receptor are transmitted through the associated DAP10 dimer (Figure 7) (59) because NKG2D lacks a tyrosine-based inhibitory motif in its cytoplasmic tail (4, 61).

Natural killer group 2 member D is expressed by all human NK cells, γδ T lymphocytes, αβ CD8^+^ T lymphocytes (6), interferon-producing killer DC (62), invariant NKT cells cells, and a small subset of effector or memory CD4^+^ T cells (4, 52, 63). Expression of NKG2D on NK cells and CD8^+^ T cells can be modulated by cytokines due to their effects on transcription and posttranscriptional processing of *NKG2D* and *DAP10*. In humans, IL-2, IL-7, IL-12 (64), IL-15, and IFN-α (65) upregulate *NKG2D* expression, whereas TGFβ (65–67), IFNβ1 (68), and IL-21 (69), IL-4, IL-12, and IFNγ (65) downmodulate *NKG2D*. This downregulation can also be attributed to the overexposure to soluble or membrane-bound NKG2DLs, which promote the internalization and subsequent degradation of the receptor or catabolites produced on macrophage activation [reactive oxygen species (ROS) and l-kynurenine] (65). This is a possible explanation of the mechanism of oxidative stress, which is a common feature of chronic renal failure. ROS trigger the upregulation of MICA and downregulation of NKG2D in NK cells *in vitro* (70). DAP10 availability is also a decisive factor in NKG2D surface expression, and miRNAs can downregulate NKG2D expression in NK cells, reducing its cytotoxic effect (65).

Fernandez-Sanchez et al. (65) have shown for the first time that epigenetic mechanisms are involved in the regulation of NKG2D expression. They analyzed the region around the translation initiation site of the *NKG2D* gene (which included 11 CpG sites between −992 and +263 positions), and they found the greatest differences in DNA methylation patterns between the positions −992 and −255. These CpGs were highly methylated in Jurkat, HUT78 cell lines and CD4^+^ T cells, partially methylated in CD8^+^ T lymphocytes and NK cells, and fully demethylated in NK cells lines. They discovered that the acetylation of histone H3 lysine 9 (H3K9) is important for correct NKG2D expression in NK and CD8^+^ T cells, while DNA demethylation may be associated with an increased expression of NKG2D in CD4^+^ T cells. The DNA methylation profile of *DAP10* gene was also analyzed, but no differences were found. CD4^+^ T lymphocytes and T cell lines (Jurkat and HUT78) had a DNA methylation; instead NKG2D-positive cells (CD8^+^ T lymphocytes, NK cells, and NKL cell line) had an unmethylated *NKG2D* gene and high levels of histone H3 lysine 9 acetylation (H3K9Ac). It was observed that the histone acetyltransferase inhibitor, curcumin, reduced H3K9Ac levels in the *NKG2D* gene, downregulated NKG2D transcription, and led to a marked reduction in the NKG2D-mediated lytic capacity of NK cell lines (65).

Another interesting study by Karimi et al. (71) of human primary NK and CD8^+^ T cells discovered a novel splice variant of human NKG2D that encodes a truncated receptor lacking the ligand-binding ectodomain (NKG2D^TR^). Overexpression of this truncated isoform severely attenuated cell killing and IFNγ release mediated by full-length NKG2D (NKG2D^FL^). A specific knockdown of an NKG2D^TR^ isoform enhanced NKG2D-mediated cytotoxicity, suggesting that NKG2D^TR^ is a negative regulator of NKG2D^FL^. At the biochemical level, it was demonstrated that NKG2D^TR^ bound to DAP10 and interfered with the DAP10–NKG2D^FL^ interaction. In addition, NKG2D^TR^ formed heterodimers with NKG2D^FL^ and negatively modulated NKG2D^FL^ preventing its surface expression. Therefore, NKG2D^TR^ constitutes a mechanism for regulation of NKG2D-mediated function in human CD8^+^ T cells and NK cells (71).

Unlike CD8^+^ T cells, TCR-mediated activation is not sufficient to induce NKG2D expression on CD4^+^ T cells, and the factors responsible for induction of NKG2D on CD4^+^ T cells are still unknown (71).

Saez-Borderias et al. (63) provided the first evidence that a subset of human cytomegalovirus (HCMV)-specific CD4^+^ T cells displays NKG2D. Their data suggest that CD4^+^NKG2D^+^ cells expanding in HCMV-stimulated cultures correspond to virus-specific memory T cells that have acquired NKG2D while losing CD28 (63).

### Conclusions on NKG2D

The *NKG2D* gene can be split into two haploblocks: *HNK1* and *LNK1* (high and low cytotoxic activity related). The NKG2D protein is a homodimer associated with two DAP10 molecules and can interact with MICA. In NK cells, the NKG2D protein is an activation receptor which is able by itself to trigger cytotoxicity. This is the main reason why it is interesting to study the relationship between MICA and NKG2D in depth in the following paragraphs.

## MICA Ligand and Its Receptor NKG2D: Functional Interactions

The crystal structure of the MICA–NKG2D complex shows that NKG2D binds to one MICA molecule as a homodimer. One of the NKG2D molecules binds mostly to the α1 domain of MICA, while the other binds mostly to the α2 domain (6). The contact between these two molecules creates a small pocket (roughly 6 Å wide × 6 Å thick × 14 Å long) (42).

The NKG2D homodimer overlays MICA diagonally in way that resembles αβTCR overlaying MHC I molecules. The central section of the MICA α2 domain is disordered when MICA is crystallized in isolated form, but it becomes ordered when MICA is bound to NKG2D and forms part of the interface between the two molecules (6).

MICA glycosylation was not essential, but it enhanced complex formation with NKG2D. Likewise, the glycosylation state of NKG2D had no substantial effect on complex formation (36).

MICA–NKG2D is considered a versatile ligand–receptor pair. As a matter of fact, NKG2D can act as a primary receptor or costimulatory molecule during infections, autoimmunity, or antitumor immune responses (6). For example, it has been shown that endothelial MICA triggeres an activating signal in allogeneic polyclonal NK cells through the immunoreceptor NKG2D, which may have account for a significant part in EC lysis by allogeneic NK cells. *In vitro* coculture assays show that interaction of endothelial MICA with NKG2D provides an immune suppressive pathway by downregulating NKG2D on the NK cell surface (14).

Boukouaci et al. (72) suggested that endocytosis of the NKG2D receptor, upon binding to sMICA, is considerably more rapid than the replenishment of cell surface NKG2D by *de novo* synthesis. The same authors also found that sMICA down regulates NKG2D receptor expression on CD8^+^ T cells. sMICA upregulates the IFNγ production only by cytokines-activated NK cells, while it has no effect on non-activated cells. The researchers demonstrated that sMICA upregulates IFNγ expression by IL-12/IL-18-activated CD3 CD56^+^ NK cells, demonstrating the pro-inflammatory effect of sMICA (72). A study with a mouse model found that Lewis rat hearts transplanted into BALB/c mice developed typical acute rejection (AR) in 6 days. The severity of xenograft rejection increased with time, from 2 to 6 days. Also increasing over time, the MICA protein and MICA mRNA reached their highest value after 6 h. The prevalence of anti-MICA was significantly higher among mice with severe AR. However, sMICA was significantly increased during AR at 2 h, then gradually decreased, and reaching its lowest value after 6 h (73).

## MICA–NKG2D and Kidney Transplant

In the last few decades, the role of MICA and NKG2D in kidney transplants has emerged (Table 4). The involvement of NK cells was discovered in 1995 when some indirect evidence was reported during rejection of kidney transplants. Accumulation of CD56^+^ NK cells expressing granzyme in kidney biopsies of patients undergoing AR suggested a role of their cytolytic activity in kidney-allograft rejection (74). Over the years, the association between NK cells and the mechanisms of microcirculation injury during antibody-mediated rejection (AMR) in kidney transplants has become increasingly evident. The researchers proposed that donor-specific antibodies (DSA) were able to bind to the endothelium and to recruit NK cells that produce IFNγ and trigger antibody (Ab)-dependent cellular cytotoxicity (75).

**Table 4:** **Relevant published work regarding NKG2D, MICA, and kidney transplants**.

Reference	Summary	MICA biomarker
**Relevant published works regarding MICA and transplants**		
Zwirner et al. (76)	Several patients had specific antibodies (Abs) against MICA. Most of them were detected in serum samples collected at different times after organ rejection	Yes

Hankey et al. (77)	MHC class I chain-related expression was documented in allografted kidneys and pancreas. Expression of MICB was observed in epithelial cells in allografted kidney and pancreas that showed histologic evidence of rejection and/or cellular injury	Yes
Opelz (78)	This work showed that non-HLA immunity contributed substantially to long-term kidney transplant failure. The targets for Abs causing late rejections could be called minor histocompatibility antigens	Yes

Mizutani et al. (79)	Patients who rejected transplants had anti-HLA and anti-MICA Abs more frequently than those with functioning grafts. These Abs found in the peripheral circulation were not necessarily donor-specific, but their association with failure was consistent with a causality hypothesis	Yes

Amezaga et al. (80)	Anti-MICA Abs were not detected pretransplant nor posttransplant in patients receiving a compatible graft. Anti-MICA Abs were detected posttransplant acute antibody-mediated rejection in patients receiving an incompatible graft	Yes

Mizutani et al. (81)	Anti-HLA and anti-MICA Abs were present independently on a more frequent basis in patients with failed grafts than those with functioning grafts	Yes

Panigrahi et al. (82)	Patients who developed both anti-HLA and anti-MICA Abs rejected their grafts more frequently than those having either of these Abs	Yes

Zou et al. (83)	Pre-sensitization of kidney transplant recipients against MICA antigens had been associated with an increased frequency of graft loss and might contribute to allograft loss among recipients who were well matched for HLA	Yes

Seiler et al. (62)	Unlike previous reports, in this work the researchers could not detect elevated MICA mRNA levels in kidney biopsies derived from patients undergoing acute rejection (AR) or chronic allograft nephropathy. In contrast, they observed a strong NKG2D mRNA induction during renal-allograft rejection, which was verified by immunohistology in kidney biopsies	No

Suarez-Alvarez et al. (84)	Anti-MICA Abs were detected in 17.6% of the patients and correlated with the development of AR. The presence of anti-MICA Abs could be an important marker for diagnosis because of their contribution to the outcome of the graft, regardless of presence of anti-HLA Abs	Yes

Alvarez-Marquez et al. (85)	At the time of the biopsy, 21% patients had only anti-HLA I Abs, 15.8% had anti-GSTT1 Abs, 10.5% had anti-HLA II Abs, and 10.5% had anti-MICA Abs. Besides anti-HLA Abs, donor-specific Abs against MICA and GSTT1 antigens could be responsible for the occurrence of Ab-mediated kidney graft rejection	Yes

Racca et al. (86)	This work did not show a correlation between MICA expression and renal graft state. The state of kidney allograft could be measured by using HLA-G1 isoforms, but not MICA mRNA levels, as markers	No

Lemy et al. (87)	The comparison between anti-MICA Abs^+^ and anti-MICA Abs^−^ patients showed that the incidence of AR episodes during the first year was similar in both groups. MICA Abs did not adversely affect renal graft outcomes	No

Li et al. (88)	Anti-MICA Abs were detected in 11 of the 15 transplant patients, irrespective of interval acute graft rejection. Also, integrative genomics predicted localization of the MICA antigen on the glomerulus in the kidney. MICA localization may explain both immunoregulatory and pathogenic roles for MICA after transplantation	Yes

Luo et al. (89)	HIF-1α plays a very important role in upregulating MICA expression and enhancing natural killer (NK) cell cytotoxicity toward target cells during hypoxia/reoxygenation in HK-2 cells. Their results demonstrated that hypoxia/reoxygenation-promoted MICA expression on HK-2 cells is through a HIF-1 pathway	Yes

Cox et al. (90)	Anti-MICA and anti-HLA Abs significantly associated with AR and anti-MICA donor-specific antibodies (DSA) and anti-HLA DSA correlated with decreased graft function by univariate and multivariate analysis. The researchers concluded that mismatching for MICA epitopes in renal transplantation is a mechanism leading to production of MICA Abs that associate with AR and graft dysfunction	Yes

Narayan et al. (91)	Case report: this case demonstrated that donor-specific anti-MICA Abs could be associated with both acute antibody-mediated rejection (AMR) and type IIA acute cellular rejection and emphasized the necessity of treating both humoral and cellular components of the rejection	Yes

Yao et al. (92)	The authors proved that Anti-MICA Abs^+^ rate was significantly higher in sensitized recipients and it had significant effect on the recovery of allograft function in early postoperative period. Protein A immunoadsorption plays an important role in decreasing preexisting Abs, especially the anti-MICA Abs	Yes

Zhang et al. (93)	Anti-MICA Abs were present in 28.9% of patients and they were associated with renal-allograft deterioration. The researchers concluded that, besides anti-HLA Abs, the presence of posttransplant anti-MICA Abs was associated with poor graft outcome and increased the risk of graft failure	Yes

Lemy et al. (94)	Anti-MICA Abs^+^ patients were more frequently anti-HLA Abs sensitized and regrafted. Four-year death-censored graft survival was not different between MICA^+^ and MICA^−^ patients. These data did not support an independent pathogenic role for MICA in long-term renal graft injury	No

Li et al. (95)	The levels of the peak mean fluorescence intensity of MICA Abs in patients with impaired renal function were significantly higher than those in normal renal function controls. They also concluded that some MICA Abs might be more important than others in mediating graft rejection	Yes

Seyhun et al. (96)	Anti-HLA class II and anti-MICA Abs^+^ were only important predictors of graft failure when present together with anti-HLA I Abs^+^. Patients who developed anti-HLA Abs alone or both anti-HLA Abs and anti-MICA Abs rejected their grafts more frequently than Abs^−^ recipients	Yes

Rodriguez Ferrero et al. (97)	They compared patients with versus without preformed circulating antibodies (circulating anti-MICA Abs and anti-HLA Abs), and they did not observe a significant difference in graft survival or renal function at 3-month follow-up	No

Solgi et al. (98)	This research supported the idea that monitoring of anti-HLA and anti-MICA Abs as well as soluble CD30 levels early after transplant had predictive value for early and late allograft dysfunctions and the presence of these factors was detrimental to graft function and survival	Yes

Akgul et al. (99)	In this study, the scientist observed the role of anti-HLA II Abs in the development of chronic active AMR and in long-term allograft survival. It is observed that anti-MICA and anti-GSTT1 Abs showed no effect on rejection mechanisms	No

Chaudhuri et al. (100)	Anti-MICA and anti-HLA Abs appeared in approximately 25% of unsensitized pediatric patients, placing them at greater risk for acute and chronic rejection with accelerated loss of graft function	Yes

Ding et al. (101)	When comparing patients with acute graft rejection against recipients with stable renal functions, the researchers highlighted a significantly higher positivity rate of anti-MICA Abs. The status of anti-MICA Abs can predict the occurrence and treatment outcomes of AR, and affect the long-term survival of the renal grafts	Yes

He et al. (102)	By following transplantation recipients during follow-ups, anti-HLA and anti-MICA Abs expression was proven to have a predictive value for early and late allograft dysfunction. The presence of donor-specific Ab is detrimental to graft function and graft survival	Yes

Jin et al. (103)	They observed the prevalence of panel-reactive antibody (PRA) and anti-MICA Abs to be increased among Ptc, albeit not significantly different from C4d AR. These results implied that Ptc could be an early indicator of AR	Yes

Li et al. (104)	CD19^+^ B cells and CD19^+^CD27^+^ memory B-cell subsets were detected from peripheral blood mononuclear cells obtained from six anti-MICA-sensitized kidney recipients. Kidney recipients had a higher percentage of CD19^+^CD27^+^ B cells compared with healthy controls. This study thus showed that B cells may be stimulated to secrete Abs	Yes

Sanchez-Zapardiel et al. (105)	The researchers detected that pretransplantation sensitization against anti-MICA and anti-HLA Abs were independent events. Preformed anti-MICA Abs independently increase risk for kidney rejection and enhance the deleterious effect of PRA^+^ status early after transplantation	Yes

Tonnerre et al. (106)	The researcher found that individual carrying *MICA A5.1/MICA A5.1* had 10-fold higher levels of *MICA* mRNA and MICA proteins at the endothelial cell surface. They also demonstrate a significant association between D/R MICA A5.1 mismatch and anti-MICA alloimmunization, particularly when donors carry the A5.1 mutation. They concluded that A5.1 mutation is an immunodominant factor and a potential risk factor for transplant survival	Yes

Zhang et al. (107)	5 years after transplantation, the frequencies of *de novo* anti-HLA and anti-MICA Abs were 25.8 and 12%, while 26.5% of patients had proteinuria. All of these factors have been associated with poor graft survival	Yes

Sapak et al. (108)	The researchers did not prove a complete correlation between the recipient anti-MICA Abs specificities and MICA antigens of the donor. They assumed that anti-MICA Ab induction occurred not only due to the allogeneic stimulation itself but also due to other factors that needed to be elucidated	No

Ming et al. (109)	Case report: the patient’s HLA alloantibodies were not specific to the first kidney donor, but the MICA alloantibodies were. This indicates the importance of MICA virtual crossmatch in the process of selection for the kidney donor if the recipient is sensitized.	Yes

Xu et al. (110)	Serum anti-HLA II Abs, anti-MICA Abs, and anti-HLA plus MICA Abs all statistically increased in renal-transplanted recipients	Yes

Cai et al. (111)	Transplant recipients had Abs against denatured HLA class I, II, and MICA antigens. However, only C1q-fixing Abs were associated with graft failure, which was related to AMR	Yes (only for c1q-fixing denaturated MICA Abs)

Sanchez-Zapardiel et al. (112)	Occasionally, preformed anti-MICA Abs may be cytotoxic by activating and fixing complement. This could lead to a reduced function in early kidney grafts	Yes

**Relevant published works regarding NKG2D and transplant**		
Feng et al. (113)	Ischemia/reperfusion injury (IRI) caused mRNA expression of Rae-1 and protein expression of Rae-1 in ischemic kidneys. This study suggested that the expression of the NKG2D ligand, Rae-1, may play a potential role in innate immunity associated with IRI	

Zheng et al. (114)	The absence of enhancement of NKG2D expression in the kidney in AN in immunodeficient mice suggested that the populations expressing NKG2D were likely to be CD8 or γδ T cells, which were not present in the immunodeficient mice, rather than macrophages, which were present and activated in both models of AN	

Seiler et al. (62)	Unlike previous reports, in this paper, the researchers could not detect elevated MICA mRNA levels in kidney biopsies derived from patients undergoing AR or chronic allograft nephropathy. In contrast, they observed a strong mRNA induction of NKG2D during renal-allograft rejection, which could be verified by immunohistology in kidney biopsies	

Hadaya et al. (115)	The results of this paper have shown an expansion of the NKG2D^+^ NK cell population during acute cytomegalovirus (CMV) infection (after kidney transplantation), which decreased over time to a level very similar to that of the control group. This suggests that the NKG2D receptor could play a similar role in NK and CD4^+^ T cells	

Zhang et al. (116)	In this study, the researchers demonstrated for the first time that NK cells could induce kidney TEC death *in vitro* and that NKG2D and Rae-1 interactions played a critical role in this killing in mice	

Shabir et al. (117)	Cytotoxic CD4^+^ CD28^null^ cell is an important biomarker for and potential mediator of adverse events after kidney transplantation. NKG2D represents an integral component of CMV immunosurveillance and immunoevasion and was upregulated on CD4^+^ CD27^−^ CD28^null^ cells isolated from patients of this study. The researchers proposed it as an important component of the cytotoxic effects (either protective or pathogenic) of these cells	

*“Yes” and “No” labels have been used if, in the studies analyzed, MICA has been valued as a possible biomarker (“Yes”) or not (“No”)*.

### NK Cells and Kidney Damage in Mice and Cell Lines

Natural killer group 2 member D-ligand engagement delivers a strong dominant activating signal that overrides the inhibitory signal delivered by self-MHC class I, thus activating NKG2D-expressing cells, resulting in innate and adaptive immunity activation (113).

Zhang et al. (116) reported a study on ischemia/reperfusion injury (IRI) on mice and discovered the capacity of NK cells to injure renal tubular epithelial cells *in vitro*. *In vivo* data supported the hypothesis that NK cells interact with tubular epithelia through NKG2D/Rae-1 interaction to mediate kidney damage following IRI.

Luo et al. (89) performed an *in vitro* study on human renal proximal tubular epithelial cell line (HK-2). They discovered that hypoxia-inducible factor-1-α (HIF-1α) plays a very important role in upregulating MICA expression and enhancing NK cell cytotoxicity toward target cells during hypoxia/reoxygenation in HK-2 cells. HIF is a heterodimer consisting of an α-subunit (HIF-1α) and a β-subunit (HIF-1β), the HIF-1β protein is constitutively present, while HIF-1α has a unique O_2_-dependent degradation domain, which leads to its degradation under normoxia conditions. The authors speculate that HIF-1α upregulates the surface expression of MICA on grafts during renal IRI, causing NK cells cytotoxicity against the organ (89).

### Possible Causes of End-stage Renal Disease

A 2009 study of the possible causes of end-stage renal disease (ESRD) (70), while note directly related to kidney transplants, inevitably reported findings of consequences for kidney transplantation. Peraldi et al. evaluated seven patients with ESRD that were treated with peritoneal dialysis, and not with the hemodialysis procedure; NKG2D expression on NK cells was significantly decreased in these patients compared to healthy donors, indicating that reduction in NKG2D expression was independent of the dialysis procedure and linked with chronic renal failure. The authors also discovered that oxidative stress in presence of increased ROS production is one of the most significant consequences of chronic renal failure, alone or in concert with other mediators, and it seems to decrease the NKG2D levels on NK cells in ESRD and to favor the upregulation of MICA expression (70).

### Anti-MICA Abs and Rejection

Some mechanisms have been proposed for MIC-mediated organ rejection. MICA antigens expressed in the allograft could induce the generation of anti-MICA Abs, which in turn might injure cells in the presence of complement.

This section contains no works that focus solely on NKG2D since most of the manuscripts are almost exclusively conserved with anti-MICA Abs: NKG2D is often just a side note; its presence and the link with MICA are given.

#### MICA-Sensitized Kidney Recipients and Higher Percentage of CD19^+^CD27^+^B Cells

CD19^+^CD27^+^ B cells are the subset of memory B cells that have the potential ability to secrete Abs. Li et al. (104) assessed the serum from 68 long-term survival kidney recipients and found 11 subjects who were MICA positive. They analyzed six MICA-sensitized kidney transplant recipients and six healthy volunteers who did not receive a transplant (control group). Healthy controls had a higher percentage of CD19^+^CD27^−^ in PBMCs than transplant patients, while the percentage of CD19^+^CD27^+^ in B cells was higher in transplant patients. The MICA-sensitized transplant patients had a significantly lower average percentage of CD19^+^ B cells in PBMC than healthy controls (3.58 ± 0.80 versus 8.53 ± 1.04%; *P* < 0.01). These results suggest that CD19^+^CD27^+^ B cells from sensitized patients have the potential ability to secrete Abs. In the same study, PBMC cells were isolated and cultured and stimulated with different molecules [toll-like receptor-9 ligand ODN-2006 CpG, PMA, B-cell activating factor (BAFF), CD40 ligand (CD40L), human recombinant IL-2 (rhuIL-2), rhuIL-10, rhuIL-4, rhuIL-21, CD40L, and BAFF] including MICA antigens. After stimulation, B cells from healthy controls and transplant patients had a lower percentage of apoptosis than non-stimulated cells. The average percentage of apoptosis cells from transplant patients was significantly higher than from healthy controls, and the IgM production (the first Ab produced by B cells after antigen stimulation) was higher in stimulated B cells from transplant patients than from healthy controls. The authors speculate that the B-cell population may be compromised by the transplant because patients are under immunosuppressive regimens, which may alter the apoptosis of B stimulated cells compared with healthy controls. The same study also performed an *in vitro* study with drugs and found that bortezomib and mycophenolic acid could inhibit B-cell Ab secretion (104).

#### MICA Abs

Hankey et al. (77) first reported that MICA and MICB expression on epithelial cells in transplanted kidneys and pancreases with histological evidence of rejection and cellular injury played a role in allograft rejection. The study showed that in a healthy kidney there was no immunochemical evidence of MIC expression. In contrast, the majority of biopsies with histologic proof of rejection or acute tubular necrosis (ATN) showed MICA positive staining of the tubular epithelium in the proximal and distal tubules. For this reason, it was concluded that alloantibodies against MICA might play a role in allograft rejection.

Zwirner et al. (76) found that several patients who had undergone a kidney transplant had specific Abs against MICA, and most of them were detected in serum samples collected at different times after organ rejection. However, these Abs were not directed against the alleles expressed by the patients, and it was speculated that if the presence of MICA Abs was probably caused by multiple blood transfusions received by the patients while awaiting a transplant, or resulting from a pregnancy or a previous transplant (76).

Lemy et al. (87) analyzed the MICA Abs from 494 controls and 597 patients with chronic kidney disease. They found a three times higher prevalence of MICA Abs in patients with chronic kidney disease when compared with controls (14.9 versus 4.7%). Nevertheless, they speculated that even if the increase in MICA Abs prevalence among patients affected by chronic kidney disease was probably related to previous renal transplantation and transfusions. Logistic regression analysis and analysis of chronic kidney disease patients who have not been subjected to transfusions and renal transplantations suggest that the increase of urea (and other nitrogenous waste) in the blood is connected to an increase of MICA immunization. The authors also reported that MICA Abs were more frequent in men than in women, despite pregnancy being an independent risk factor for the development of MICA Abs (87). This finding is in sharp contrast with other published work. The fact that nearly one-third of MICA chronic kidney disease stage V patients have never experienced any identifiable immunizing event indicates that there must be other causes for MICA sensitization. At the same time, one-fifth of the same patients showed the presence of autoreactive MICA IgG Abs, distinctly rare with respect to HLA Abs. The authors showed that patients with MICA Abs had a somewhat better overall graft survival than MICA Abs^−^ patients. Finally, Lemy et al. found in MICA Abs^+^ and MICA Abs^−^ patients a similar incidence of AR episodes during the first year (10.2 versus 12.8%), as well as similar levels of proteinuria and creatinine (87).

Another study of MICA Abs screened 147 recipients with end-stage renal disease; 82 of these patients were Abs^+^ (55.8%). Forty patients had both anti-HLA and anti-MICA, 33 had only anti-HLA, and 9 only anti-MICA Abs in the posttransplant period. The authors found that patients who developed HLA alone, or both HLA and MICA Abs, rejected their grafts more frequently than Abs^−^ recipients. The rates of HLA class I, class II, or both Abs^+^ were greater in the rejection patients than the non-rejection patients (*P* = 0.011, 0.037, and 0.0275, respectively). So the authors speculated that HLA class II and MICA Abs^+^ were the only important predictors of graft failure when both of them were present with HLA class I Abs^+^ (96).

In a retrospective study, Solgi et al. (98) analyzed sera samples of 40 living unrelated donor kidney recipients, looking at anti-HLA and anti-MICA Abs and the levels of soluble CD30 (sCD30) and sMICA. They found that patients with pre- and posttransplant HLA Abs had a higher incidence of AR episodes (*P* = 0.01 and *P* = 0.02), more graft loss (*P* = 0.001), and lower graft survival during a mean follow-up of 3 years. This group of patients also had higher levels of sCD30 and serum creatinine and decreased contents of sMICA early after transplantation, as compared to the patients without HLA Abs. Anti-MICA Abs were observed in 8/40 (20%) and 5/40 (12.5%) of all patients pre- and posttransplant, respectively. HLA and MICA Abs were both found in two out of four cases with graft loss. In a comparison of transplant rejecting to functioning graft groups, sCD30 levels increased at day 14 (*P* = 0.001), while sMICA levels were insignificantly lower in the first group (98).

Chaudhuri et al. (100) studied the evolution of humoral immunity in low-risk pediatric patients during the first 2 years after renal transplantation. They correlated the presence of serum anti-HLA DSA and serum MICA Abs with clinical outcomes and histology (the biopsies were performed at 0, 6, 12, and 24 months). They found anti-HLA Abs in 22% of patients, 6% of which were donor-specific, while 6% developed anti-MICA Abs. Three percent of patients developed *de novo* Abs to both HLA and MICA. The presence of *de novo* Abs was associated with significantly higher risks for AR (*P* = 0.02), chronic graft injury (*P* = 0.02), and decline in graft function (*P* = 0.02). Graft function was monitored by the difference between creatinine clearances. Anti-MICA and -HLA Abs were found in 25% of unsensitized pediatric patients. This was correlated with a greater risk of acute and chronic rejection (100).

Zhang et al. (107) associated the presence of *de novo* MICA Abs and proteinuria with graft failure, after renal transplantation. They investigated 275 patients without preexisting anti-HLA and -MICA Abs. Five years after renal transplantation, 25.8% showed *de novo* anti-HLA Abs, 12% showed *de novo* anti-MICA Abs, and 26.5% proteinuria. *De novo* anti-HLA Abs were associated with increased proteinuria after transplantation (relative risk, 3.12). Anti-HLA Abs and proteinuria were both associated with poor 5-year graft survival (*P* = 0.027 and *P* = 0.006, respectively). Patients with *de novo* anti-MICA Abs were also apt to have proteinuria. The authors concluded that *de novo* anti-HLA and -MICA Abs and proteinuria are all associated with poor graft survival (107).

#### Pretransplant Panel-Reactive Abs and Preexistent Circulating Abs

Opelz (78) studied the influence of pretransplant panel-reactive antibody (PRA) status on the long-term outcome of kidney grafts from HLA-A, -B, and -DR, identical sibling donors. In over 10 years of follow-up, he discovered that non-HLA-directed immunity and Abs against HLA had a similar influence for the long-term results for kidney recipients with PRA. Opelz suggested that the targets for Abs causing late rejections could be the so-called minor histocompatibility antigens (78).

Sanchez-Zapardiel et al. (105) studied 727 transplanted patients and showed that the effect of anti-MICA Abs occurs independently of the presence of anti-HLA Abs. Pacients were categorized into four groups according to the presence (+) or absence (−) of anti-HLA and anti-MICA Abs: HLA^+^MICA^+^ (*n* = 27); HLA^−^MICA^−^ (*n* = 510); HLA^+^MICA^−^ (*n* = 165), and HLA^−^MICA^+^ (*n* = 25). A notable difference was observed 3 months after transplantation, when HLA^−^MICA^+^ patients had a graft rejection rate of 8% compared with 2% in HLA^−^ MICA^−^ patients. The patients were also grouped according to the presence of preexisting anti-HLA Abs, as measured by % PRA (PRA^+^ or PRA^−^): PRA^+^MICA^+^ (*n* = 7), PRA^−^MICA^−^ (*n* = 610), PRA^+^MICA^−^ (*n* = 65), and PRA^−^MICA^+^ (*n* = 45). The incidence of rejection was found to be superior in PRA^+^MICA^−^ cohort versus PRA^−^MICA^−^ patients (24 months after transplantation), but allograft rejection rate was the highest when comparing PRA^+^MICA^+^ patients with PRA^−^MICA^−^ patients 3 months after transplantation, a finding which was repeated at 6 months (105). This work is of interest because it performed a comparative study on the effects of anti-MICA and anti-HLA Abs on kidney transplants.

The Rodriguez Ferrero et al.’s (97) study included 22 recipients of kidney transplantations from deceased donors, and no differences between patients that showed preexistent circulating antibodies (CA) and those that did not were reported. In regards to the incidence of AR episodes, the only factor associated with CA was re-transplantation. So the authors concluded that CA monitoring is important for highly sensitized renal transplants, but they did not observe a difference in graft survival or renal function in the first 3-month follow-up (97).

#### Cd4 Deposition and C1q-Fixing Abs

A study of patients with acute antibody-mediated rejection (AAMR), who had MICA*008 Ab, showed that the presence of anti-MICA Abs and the deposition of C4d in biopsies performed at the time of AAMR was associated with the detection of DSA or Abs against HLA (80). The observation that the control group of 30 patients with long-term functioning grafts did not have anti-MICA*008 Abs provided indirect evidence of the importance of anti-MICA Abs in chronic rejection. Furthermore, all patients receiving an allograft fully matched at MICA had functioning grafts (80). It is also important to mention that MICA Abs are able to activate complement in *in vitro* experiments (80).

Alvarez-Marquez et al. (85) selected 58 patients that underwent a kidney biopsy because of primary non-function, delayed graft function or acute dysfunction of a previously functional graft, suspected by oliguria, increase of serum creatinine levels, or proteinuria. At the time of the transplant, all patients showed negative complement-dependent cytotoxicity crossmatches. Researchers demonstrated that 80% of a group of 19 patients with clinically evident graft dysfunction and with C4d deposition in kidney biopsies had Abs directed against donor-specific HLA class I, class II, MICA, or GSTT1 (glutathione-*S*-transferase T1) antigens (85).

In the Li et al.’s (88) study, a human ProtoArray platform was used to study 37 serum samples from 15 renal transplant patients (pediatric and young adult) with (*n* = 10) and without (*n* = 5) AR, and seven normal controls. To test serum Abs, they used a ProtoArray containing 5,056 non-redundant human proteins expressed in a baculovirus system, purified from insect cells and printed in duplicate onto a nitrocellulose-coated glass slide. Moreover, all patients were primary transplant recipients, and the biopsies were graded by the Banff classification. The authors found that the mean immune response signal in posttransplant patient serum showed an increase in anti-MICA Abs when compared with healthy normal controls (*n* = 7), but anti-MICA Abs signal intensity was unrelated to the sampling time interval posttransplantation. Mean MICA Abs signal intensity was higher in transplant patients with C4d^+^AR (121.4) versus C4d^−^AR (4.3), so a correlation between high MICA Abs levels and C4d^+^ graft rejection *r* = 0.54 (*P* = 0.039) was observed. On ProtoArray, each gene on the cDNA platform was compared between a specific kidney compartment versus all other compartments, by a two-unpaired class comparison and a multi-class comparison. The signal intensity of anti-MICA Abs ranked in the top 15 for glomerulus, so the MICA antigen was found to have a 2.7-fold higher expression in the glomerulus when compared to the other 6 normal kidney compartments. Cytoplasmic granular staining for MICA in normal and stable transplanted kidneys was observed solely in podocytes within glomeruli. In AR, in addition to the persisting glomerular staining, the infiltrating mononuclear lymphocytes also showed strong positive staining for MICA. So the authors demonstrated that Ab responses in patients are modulated by MICA after transplantation in patients, irrespective of graft rejection (88).

Another study correlates Cd4 deposition and creatinine levels. Ding et al. (101) evaluated serum anti-MICA Abs before and after kidney transplant, and they also examined PRA, serum creatinine, urine, graft ultrasound, lymphocyte subsets, and the pathology of graft biopsy. The study was split into two parts. In the first part, patients with AR were grouped into MICA^+^, MICA^−^ (*P* < 0.05) and control groups. There were a significantly higher number of anti-MICA Abs positive patients with acute graft rejection compared with stable renal functions patients (control group).

Two to three days after the occurrence of AR, the anti-MICA Abs level increased gradually. Anti-rejection treatment had no effect on anti-MICA Abs but lowered serum creatinine to a normal level. In the second part, the authors analyzed chronic graft rejection patients. The number of anti-MICA Ab positive patients was significantly higher than those with stable renal function (*P* < 0.05), and the serum creatinine levels were significantly higher in MICA^+^ than in MICA^−^ cases (*P* < 0.05). The authors also found that graft biopsy of all MICA^+^ cases showed C4d deposition (101).

Jin et al. (103) studied 53 cases of AR that showed C4d deposition in the peritubular capillaries, 50 cases of ARs without C4d deposition, 30 with peritubular capillaries alone, 28 with ATN, and 78 patients with surveillance biopsies (control group). The authors observed that the prevalence of PRA and anti-MICA Abs was increased among the peritubular capillaries alone group (30.0 and 43.3%, respectively), albeit not significantly different from the group with C4d^+^ AR (49.1 and 39.6%, respectively). They also observed that the immunophenotype of infiltrating T lymphocytes and serum Abs (85.9% of control biopsies presented) had a regulatory phenotype while in the peritubular capillaries cohort, 93.3% of biopsies showed the cytotoxic phenotype. These results showed that peritubular capillaries in biopsy specimens from patients with early renal-allograft dysfunction could be an indicator of AR, especially acute humoral rejection (103).

Cai et al. (111) collected samples from 975 kidney transplant recipients, and they tested for C1q-fixing Abs against denatured HLA class I, class II, and MICA antigens. Among 169 patients who lost renal grafts, 44% had c1q-fixing Abs against denatured HLA/MICA antigens, which was significantly higher in patients with functioning renal transplants (25%). They concluded that C1q-fixing Abs were significantly associated with graft failure caused by AMR (72.73%) and they affirmed that only c1q-fixing Abs were associated with graft failure and AMR (111).

#### MICA Allele Epitopes and Eplets

Regarding the anti-MICA Abs, Duquesnoy et al. (118) developed an eplet-based version of the HLA-Matchmaker algorithm as a tool to assess the epitope specificity of these Abs. A repertoire of 38 potentially immunogenic *MICA* eplets was selected (based on MICA structure molecular viewing and the amino acid sequence differences between *MICA* alleles). These eplets are based on a functional epitope structure (a configuration of amino acids within a 3 Å radius of an Ab accessible polymorphic residue on the molecular surface). In this study, the eplet frequencies were calculated from *MICA* allele frequencies in 1,245 European-Americans and 605 African-Americans. Many eplets are shared by very similar groups of *MICA* alleles. For instance, the combination of eplets called CMGWS “supereplet” is composed by 36C, 129M, 206GW, and 215S epitopes and shared by the same group of *MICA* alleles (A*001, A*002, A*007, A*011, A*012, A*015, A*017, A*018, A*021, A*030, A*041, A*043, A*045, A*046, A*047, A*014, A*020, A*023, A*026, A*029, A*036, A*040, A*050, A*052, and A*055). The random chance that these eplets are a mismatch is 20.1% in African-Americans and 24.0% in European-Americans. Alternatively, the combination of eplets named AYVE “supereplet” is composed by 25AY, 129V, and 173E and was shared by another group of *MICA* alleles (A*004, A*006, A*008, A*009, A*010, A*016, A*019, A*024, and A*044). The random chance of their being a mismatch is 28.2% in African-Americans and 20.1% in European-Americans (118).

Panigrahi et al. (82, 119) analyzed the presence of Abs against MICA*001, MICA*002, MICA*004, MICA*008, and MICA*009 in serum samples of 185 patients transplanted with live related donor kidneys. Sixteen percent of all recipients developed anti-MICA Abs during the posttransplant period, 83% of the patients whose grafts eventually failed had both anti-HLA and anti-MICA Abs as compared to 29% patients who had only anti-MICA Abs, and 11% of those without any of the Abs (HLA or MICA) (82, 119).

Analysis of anti-MICA*001, MICA*002, MICA*004, MICA*008, and MICA*009 Abs in serum samples from 1,910 kidney recipients showed that a correlation between the presence of anti-MICA Abs and the reduced in kidney-allograft survival was not influenced by the simultaneous presence of Abs against HLA (120). In this study, decreased renal-allograft survival is associated with anti-MICA Abs formed before transplantation. It was also found that patients with Abs against MICA before transplantation did not received more transfusions than patients without such Abs, in contrast with the Zwirner et al.’s study (76). So the authors speculate that cross-reactivity with substances from the environment may play a role in priming the immune system, facilitating anti-MICA Ab production (120).

Suarez-Alvarez et al. (84) screened 284 kidney transplant sera for anti-MICA Abs and mapped the epitopes of MICA by screening a library of synthetic overlapping peptides from the extracellular domains of the protein against the sera from kidney transplant patients with anti-MICA Abs. Anti-MICA Abs were detected in 50 of 284 patients (17.6%), and they correlated with the development of AR. The authors found that nine regions were reactive with anti-MICA Abs. Five epitopes were located in constant regions (II, III, IV, VI, and IX) and were present in all *MICA* alleles, while the other four regions (I, V, VII, and VIII) mapped to variable sites of polymorphic amino acids among the different alleles products of MICA. In particular, regions V, VII, and VIII were the regions with the highest amino acid variability. Three polymorphic residues, 173 (E/K), 175 (S/G), and 181 (R/T), had determined allele-specific epitopes. The aminoacid 208Y and 213T, instead, contributed in the cross-reactivity among alleles (84).

Cox et al. (90) identified MICA IgG Abs directed against MICA*001, *002, *004, *007, *008, *009, *012, *017, *018, *019, and *027. Analysis of 116 healthy control subjects revealed only one subject with anti-MICA Abs (0.9%) and five subjects (4%) with anti-HLA class II Abs, while in a subgroup of 227 transplant recipients and their donors the coproduction of Abs to HLA and MICA significantly associated with acute cellular rejection (ACR). Analysis of patients with AAMR established strong associations with the presence of Abs against HLA class I and II, but not anti-MICA. By aligning *MICA* allele profiles present in the subgroup of 227 renal graft recipients and their respective donors, it was possible to establish the precise position of amino acid mismatches that correlate strongly with MICA Ab production. Mismatching at residues 36, 129, 173, 175, 213, and 251 showed the strongest association with anti-MICA Ab production in transplant recipients, while 91, 125, 156, and 221 residues were also mismatched between recipients and donors, but were not significantly associated with anti-MICA Ab production. There are two immunodominant motifs: MICA-G1 is characterized by residues 36 cysteine (C), 129 methionine (M), 173 lysine (K), 206 glycine (G), 210 tryptophan (W), and 215 serine (S). Alternatively MICA-G2 epitopes share residues 36 tyrosine (Y), 129 valine (V), 173 glutamic acid (E), 206 serine (S), 210 arginine (R), and 215 threonine (T). The majority of these recipients (10 out of 17 individuals, 59%) developed *de novo* donor-specific anri-MICA Abs posttransplantation, and there was a significant association of graft dysfunction with the presence of anti-MICA DSA alone after 2 years. In conclusion, it was discovered that mismatching *MICA* alleles lead to the development of anti-MICA Abs in some renal graft recipients, and the presence of anti-MICA DSA was independently associated with decreased glomerular filtration rate (eGFR) and poorer graft outcome (90).

Tonnerre et al. (106) went beyond the usual studies of anti-MICA Abs and focused on searching for a specific allele that could lead to a poorer outcome. The authors performed a study that showed that the MICA*008 (A5.1) molecule is a major antigenic determinant and target for recipient sensitization of kidney transplant patients. *MICA A5.1* is associated with four alleles: *023, *028, *053, and *008. The authors divided primary EC cultures from transplant donors in *MICA A5.1* homozygous, heterozygous, and control. The MICA surface expression was significantly higher on ECs from *A5.1/A5.1* donors than from controls. The *MICA A5.1* allele also leads to a reduction of sMICA and an increase in the MICA level in exosomes in ECs. Anti-MICA (A5.1) Abs intensities in the sera of recipients with anti-MICA Abs were not higher than intensities observed for other anti-MICA (control) Abs. However, when tested on EC cultures expressing physiologic levels of membrane-bound MICA, the sera only bound to ECs from *MICA A5.1* donors. This seemed to show that anti-MICA Abs bind ECs’ targets in an allele-specific manner.

In fact, the combination of the donor carrying *MICA A5.1* and the recipient having a non-*MICA A5.1* allele was overrepresented in the group of MICA-sensitized patients compared with the group of non-immunized recipients (106).

Sapak et al. (108) concluded that anti-MICA Abs could not be responsible for the rejection if they were not directly detected in the transplanted graft. In the sera of 124 renal recipients, the authors found only 22 patients positive for anti-MICA Abs. The most frequent anti-MICA Abs were directed against MICA*018 and MICA*001. MICA*008 had the highest gene frequency (31%), followed by MICA*002 (14%). Comparing *MICA* allele profiles of donors and anti-MICA Ab epitopes of their respective recipients, Sapak et al. found a match in only in 9 donor–recipient pairs (41%) while the sera of the other 13 patients was negative for Abs against graft MICA molecules, but positive for Abs against other MICA antigens. The majority (59%) of anti-MICA Abs in patients were not donor-specific, so the authors suggested that anti-MICA Ab induction was not caused by renal graft allogeneic stimulation but was also probably stimulated by other still unknown immune mechanisms (108).

Sanchez-Zapardiel et al. (112) studied 727 kidney recipients. They found that PRA^+^MICA^+^ recipients exhibited a longer time to reach optimal serum creatinine level after transplantation (*P* = 0.005) had the lowest eGFR at 3 months and PRA^+^MICA^+^ status independently increased the risk for chronic kidney disease stage 5 at month 3. Pretransplant anti-MICA Abs were poly-specific; anti-AYVE supereplet reactivity was higher in HLA^+^MICA^+^ versus HLA^−^MICA^+^ patients and superior than anti-CMGWS supereplet within HLA^+^MICA^+^ patients. The authors also found that some preformed anti-MICA Abs might bind complement, using the C1q Luminex assay. Sanchez-Zapardiel et al. analyzed 13 anti-MICA^+^ pretransplant sera that were positive for the C1q binding assay and one of them (serum 3) exclusively recognized the AYVE supereplet with a strong reactivity against MICA*027 antigen. The authors concluded that these preformed anti-MICA Abs are able to mediate cell death by fixing and activating the complement cascade. So they speculated that the anti-MICA Abs might contribute to worse early kidney graft function (112).

#### Correlation between Anti-MICA Abs and Creatinine Levels or Estimated Glomerular Filtration Rate (eGRF) or Death-Censored Graft Survival (DCGSs)

Yao et al. (92) included 29 sensitized recipient patients who had undergone living-related donor renal transplantation between 2007 and 2009. They found a statistical difference in postoperative serum creatinine levels within 1 week between anti-MICA Ab-positive (135.4 ± 21.4 mol/L) and anti-MICA Ab-negative groups (108.6 ± 31.6 mol/L), but no significant difference between the two groups at discharge. To decrease the preexisting Abs (mainly IgG, IgM, and IgE), all recipients were treated with protein A immunoadsorptions, and this therapy was effective in decreasing anti-MICA Abs (92).

Zhang et al. (93) studied patients receiving primary kidney transplants (all from deceased donors) between 2004 and 2007. No significant association was found between the presence of anti-MICA and -HLA Abs, nor between the presence of anti-MICA Abs and 1-year graft survival rate. However, during the follow-up period, eGFR decreased 24.0 ± 3.4% in the anti-MICA Abs positive group, while it decreased only 8.4 ± 3.0% in anti-MICA Abs negative patients. A strong correlation between the production of anti-MICA Abs and renal impairment was also found. For these reasons, the authors concluded that patients with anti-MICA Abs had a more rapid deterioration of graft function, compared to those without anti-MICA Abs (93).

In another study that did not recognize MICA as a biomarker, sera from 779 kidney transplant recipients was tested with two single-antigen flow bead assays 1 year after transplantation. Thirteen of the 779 patients were lost to follow-up, 50 had lost their graft, and 33 died with a functioning graft. The prevalence of anti-MICA Abs was 5.3% at 1-year posttransplantation, and that MICA^+^ patients were more frequently HLA sensitized and regrafted. However, 4-year DCGSs were not different between MICA^+^ and MICA^−^ patients (97 versus 94%, *P* = 0.28), and 4- and 8-year survival rates were similar in MICA^+^ and MICA^−^ patients. Thus, the hypothesis of an independent pathogenic role for MICA in long-term renal graft injury was not supported, and the authors questioned the utility of monitoring anti-MICA Abs posttransplant with single-antigen flow bead assays (94).

#### MICA Abs in Case Study

Narayan et al.’s (91) case study focused on a 14-year-old girl with branchiooto renal syndrome who underwent re-transplantation with an HLA crossmatch-negative deceased donor kidney. She lost her first kidney transplant to chronic rejection at the age of 10 and underwent allograft nephrectomy. She was highly sensitized, and to improve her chances for transplantation, she underwent desensitization with high-dose IVIG and rituximab. When she received a deceased donor renal transplant, the pretransplant anti-HLA Ab testing showed no anti-donor HLA Abs. The patient maintained good allograft function until postoperative day 10 when she presented with fever and anuric renal failure. The only Ab found was donor-specific anti-MICA Ab, specifically directed against MICA*012 protein. Evaluation of the pretransplant serum revealed preformed anti-MICA*012 Abs with levels that were elevated both before transplant and at the time of rejection. Anti-MICA Abs levels declined with the initiation of plasmapheresis and IVIG and correlated well with normalization of renal function and resolution of ACR and AMR. The authors speculated that the sensitization to the MICA*012 protein was caused by prior sensitization from the first renal transplant or previous infections or transfusions. The conclusion of their research is that donor-specific anti-MICA Abs can be associated with both AMR and Banff type IIA ACR and may require treatment with plasmapheresis (91).

Ming et al. (109) studied a patient who suffered early aggressive AMR in the presence of DSA against MICA after her first renal transplant. The researchers found that anti-MICA–DSA in recipient serum could bind MICA-G1 antigens expressed in the cultured human umbilical cord vein endothelial cells (HUVECs). The recipient serum was cytotoxic to these HUVECs, but not against HUVECs that did not express MICA-G1 antigens in the presence of complement. The researchers discovered that the patient had been sensitized to MICA antigens and HLA, before transplantation, and the HLA alloantibodies were not specific to the first kidney donor, but the MICA alloantibodies were. In light of this discovery, the second renal transplant was with a negative MICA virtual crossmatch, and it was successful (109).

### microRNA and mRNA’s Analysis

Seiler et al. (62) showed that an elevated NKG2D mRNA expression in biopsy material was correlated with the severity of AR and detected NKG2D^+^ cells located in clusters around tubules in biopsies derived from patients diagnosed with acute and chronic rejection. The expression of *NKG2D* mRNA was also detected in urinary sediments obtained 2–3 days before the AR episode. However, significant levels of MICA mRNA were not detected in the patient groups analyzed (62). For the first time, the focus was on the importance of the role of the NKG2D molecule, which is responsible for MICA signal transduction.

Another controversial paper regarding the role of MICA is the Racca et al.’s study (86), in which the authors obtained peripheral blood samples from 29 renal-transplanted patients (19 men). They classified patients it into three groups: AR group (9 patients with acute grade I/II allograft rejection), chronic rejection group (10 patients with chronic allograft rejection), and stable evolution group (10 patients with clinically stable allograft evolution). The authors observed that MICA mRNA levels in peripheral blood mononuclear cells showed similar expression levels in all groups evaluated and in the control group. They also found similar levels of MICA expression in a comparison of biopsy specimens from AR and nephrotoxic ATN patients. They did not find a correlation between MICA expression and renal graft state (86). It is interesting to note that the MICA expression in biopsies did not have a healthy control group, while expression of MICA mRNA may be a posttranscriptional control that modules MICA expression on the cell surface. The Racca et al.’s (86) study still represents an interesting opportunity to discuss the role of MICA as a biomarker.

Xu et al. (110) studied miR-338-5p, a microRNA downregulated in AMR renal allografts, and negatively correlated with BAFF. This molecule plays an important role in the differentiation, development, and proliferation of B lymphocytes. BAFF could be released in a soluble form (sBAFF) after cleavage and would bind to BAFF receptor. The receptor-associated factor 3 is a sort of adaptor for the BAFF–BAFF-R connection, it is implicated in a signal transduction, and it appeared to be a candidate target for miR-338-5p. In the study, 49 follow-up renal-transplanted recipients and a healthy control group were examined, and it was found that anti-HLA II Ab, anti-MICA Ab, and anti-HLA + MICA mixed Abs were all statistically increased in recipients. Serum miR-338-5p was significantly downregulated in renal-transplanted recipients compared with healthy volunteers and was inversely correlated with sBAFF. The authors speculate that miR-338-5p may regulate the BAFF signal, and they suggested that sBAFF was significantly negatively correlated with anti-MICA Abs (110).

### Cytomegalovirus (CMV) and Polyomavirus and Transplantation

Cytomegalovirus infection is the most common viral complication after renal transplantation and solid organ transplantation in general. One hundred ninety-six recipients who underwent kidney transplantation during the past 6 years were assessed with at follow-up of at least 12 months. In this study, it was shown that the activating receptor NKG2D was expressed in a significantly higher number of NK cells at day 0 and day 20 compared to day 180 (*P* = 0.01 and *P* = 0.003, respectively) and compared to the control group (*P* = 0.0003 and *P* = 0.0004, respectively) (121). This finding suggests a possible mechanism for the activation of NKG2D that goes beyond organ rejection, but it is closely related. In fact, in the Hadaya et al. (121) study, it was shown that an expansion of the NKG2D^+^ NK cell population occurred during acute CMV infection which decreased over time to a level very similar to that of the control group.

An interesting study that involved NKG2D, performed by Shabir et al. (117), demonstrated that CD4^+^CD28^null^ T cell expansion is driven by latent CMV infection inflammation. The immune surveillance of CMV may have an unwanted consequence in the development of endothelial injury, which was proven to be mediated by CD4^+^CD27^−^CD28^null^ cells in *in vitro* experiments. NKG2D was upregulated on CD4^+^CD27^−^CD28^null^ cells isolated from patients in this study and might have an important component of the cytotoxic effects of these cells. In fact, CD4^+^CD28^null^ cells were found predominantly in CMV-seropositive patients, and expanded in the posttransplantation period, and expressed markers of cytotoxicity (NKG2D and perforin) and endothelial homing (CX3CR1). Isolated CD4^+^CD27^−^CD28^null^ cells previously exposed only to CMV-derived antigens showed signs of endothelial damage and apoptosis, and this effect was mitigated by NKG2D-blocking Ab. They concluded that the increase in CD4^+^CD28^null^ cell frequencies was associated with delayed graft function and lower eGRF at end follow-up, and this could be mediated by NKG2D (117).

Another study by Tonnerre et al. (122) investigated the implication of MICA in BK polyomavirus (BKPyV) reactivation in a cohort of 144 transplant donor/recipient pairs including recipients with no reactivation (control). BKPyV is frequently reactivated in kidney transplant recipients receiving an immunosuppressive regimen and is associated with nephropathy (BKPyVAN) and graft rejection. They investigated the impact of the *MICA A5.1* mutation on recipient BKPyV reactivation, and they found that recipients carrying a *non*-*MICA A5.1 (nA5.1)* genotype transplanted with a kidney from a donor carrying the *A5.1 MICA* variant had a lower risk of BKPyV reactivation (*P* = 0.0148). So they speculated that *MICA A5.1* could be a protective allele toward BKPyV infection (122). Interestingly, these researchers also found that the donor (A5.1)–recipient (nA5.1) combination was overrepresented in the group of MICA-sensitized patients, but in the latter, MICA A5.1 seemed to be a protective factor for a virus related to graft rejection (106).

## Conclusion

Since the *MICA* gene was first described, it has been the subject of many studies aiming to comprehend its immunobiology and the role it plays in fine-tuning the innate and adaptive immune response. MICA appears to be involved in transplant rejection, immune response against viruses and intracellular bacteria, inflammation, homeostasis of epithelia, immune response against tumors, and tumor immune escape mechanisms. However, there remain a number of open issues to be addressed surrounding MICA’s functions and roles. Developing and implementing typing strategies for *MICA* alleles may increase the chance for positive outcomes in solid organ transplantation by allowing better matching. MICA’s biological function is achieved through its interaction with the NKG2D receptor. This activating receptor and its ligands are deeply involved in the outcomes of transplanted grafts, in fact, the overexpression of NKG2DLs could be involved in rejection episodes and can contribute to graft loss (44).

Various studies have shown that anti-MICA Abs, binding to MICA molecules expressed at the endothelial allograft cell surface, may have relevance to kidney transplantation outcome (81, 106). However, it is important to note that some studies, such as that of Lemy et al. (87), where the presence of anti-MICA Abs do not show adverse effects in renal graft outcomes (87). Also, *MICA* mRNA level analysis in blood mononuclear cells did not show a correlation between *MICA* expression and renal graft state (86). Seiler et al. (62) did not find an enhancement of mRNA expression levels of MICA in kidney biopsies from patients undergoing AR or chronic allograft nephropathy, but they observed increased *NKG2D* expression. In an interesting study performed by Sapak et al. (108), 41% of the detected anti-MICA Abs were donor-specific, but an astonishing 36% were anti-MICA Abs against self-MICA antigens and several patients (27%) produced both (108).

Regarding NKG2D, there are studies that report that it is possible to prolong graft survival and to prevent CD28-independent rejection of cardiac allografts after blocking NKG2D (123).

We can conclude that the role of MICA and NKG2D in transplant outcome is not yet clear; MICA-mediated rejection probably is not just a reaction to the MICA non-self protein. The stress condition following a transplant causes a general inflammatory status in the recipient. This could increase MICA production, thus activating the response *via* the NKG2D receptor. The clinical impact of these interactions will remain unclear until further studies are performed.

## Author Contributions

MR: planning and organizing structure of the review; research and analysis of the papers; wrote the review; and planning and creation of figures and tables. MB: planning and organizing structure of the review and contributions to the sections writing/critical review of the manuscript.

## Conflict of Interest Statement

The authors declare that the research was conducted in the absence of any commercial or financial relationships that could be construed as a potential conflict of interest.

## Footnotes

^2^https://www.ncbi.nlm.nih.gov/gene?Db=gene&Cmd=DetailsSearch&Term=100507436#.

^3^http://hla.alleles.org/nomenclature/stats.html.

^4^http://hla.alleles.org/alleles/classo.html.

^5^http://browser.1000genomes.org/Homo_sapiens/Variation/Population?db=core;r=12:10524865-10525865;v=rs1049174;vdb=variation;vf=750969.
